# Fighting poor-quality medicines in low- and middle-income countries: the importance of advocacy and pedagogy

**DOI:** 10.1186/s40545-016-0088-0

**Published:** 2016-11-10

**Authors:** Raffaella Ravinetto, Daniel Vandenbergh, Cécile Macé, Corinne Pouget, Brigitte Renchon, Jean Rigal, Benedetta Schiavetti, Jean-Michel Caudron

**Affiliations:** 1 QUAMED, Institute of Tropical Medicine, Antwerp, Belgium; 2 AEDES, Brussels, Belgium; 3 Department of Essential Medicines and Health Products, World Health Organization, Geneva, Switzerland; 4 Médecins Sans Frontières, Paris, France; 5 Independent, Brussels, Belgium

**Keywords:** Quality of medicines, Low- and middle-income countries, Pharmaceutical regulation, Quality assurance, Equity in health

## Abstract

The globalization of pharmaceutical production has not been accompanied by a strengthening and harmonization of the regulatory systems worldwide. Thus, the global market is characterized today by a situation of multiple standards, and patients in low- and middle-income countries are exposed to the risk of receiving poor-quality medicines. Among those who first raised the alarm on this problem, there were pioneering humanitarian groups, who were in a privileged position to witness the gap in quality of medicines between high-income countries and low- and middle-income countries.

Despite an increasing awareness of the problem and the launch of some positive initiatives, the divide in pharmaceutical quality between the North and the South remains important, and insufficiently addressed. More advocacy is needed for universal access to quality-assured medicines. It should target all those who are strongly “involved” with medicines: regulators, international organizations, journalists, purchasers, prescribers, program managers, policy makers, public health actors and the patients. Advocacy should be based on evidence from research and monitoring programs, and technical concepts should be translated in lay language through communication tools that address all the stakeholders. The fight to ensure universal access to quality medicines needs the participation of all, and can only be successful if grounded in common understanding.

## Background

Over the last three decades, we have witnessed an increasing globalization of pharmaceutical production. The pharmaceutical ingredients and finished products are manufactured in many different regions and countries, and they move on the international market through multiple distribution channels. Unfortunately, this shift has not been accompanied by a strengthening and harmonization of the regulatory systems worldwide [1]. As a result, the global pharmaceutical market is characterized by a situation of multiple standards of quality [2]. Many under-resourced National Regulatory Authorities in low- and middle-income countries (LMICs) lack the capacity to fully assure the quality of medicines circulating in their territory, and the most vulnerable populations are exposed to the risk of receiving poor-quality medicines [3]. For instance, it is estimated that 34 % of medicines in sub-Saharan Africa are of poor quality [4]. In the field of malaria, the analysis of a database collating customized summaries of 251 published anti-malarial quality reports showed that out of 9,348 anti-malarials sampled, 30.1 % (2,813) failed chemical/packaging quality [5]. In addition, because of the complexity of the supply channels in the global market and the consequent difficulty to trace the origin of medicines, poor-quality products may exceptionally reach high-income countries, and in a few case they have even been used in clinical trials [6].

Poor-quality medicines include substandard medicines, i.e. genuine medicines that are produced by manufacturers authorized by a National Regulatory Authority but do not meet quality specifications, and falsified or counterfeit medicines, i.e. medicines deliberately and fraudulently mislabeled with respect to identity and/or source. While in case of falsified medicines there is a deliberate (criminal) willingness to fraud, substandard medicines result from human error or negligence at manufacturing sites. Consequently, corrective actions are very different: falsified medicines can be fought by repressing illegal manufacturing and distribution, while substandard medicines may only be tackled by strengthening the capacities of National Regulatory Authorities [7]. Noteworthy, since there is currently no universally agreed definition of ‘counterfeit medicine’, the World Health Organization (WHO) has adopted since 2012 the broader wording “substandard, spurious, falsely labelled, falsified and counterfeit” (SSFFC) medical products, and this “until a new definition is agreed” (http://www.who.int/medicines/regulation/ssffc/definitions/en/). However, irrespectively of definitions, there are no differences in what concerns the risks for the patients, i.e. therapeutic failure, toxicity and/or emergence of resistance, all leading to a great deal of avoidable human suffering, including possibly death.

Among those who first raised the alarm about the weakness of pharmaceutical quality assurance in LMICs, there were pharmacists working in humanitarian medical projects [8]. They were in a privileged position to witness the gap between their own countries (at that time, mostly humanitarian workers came from Western countries) and the LMICs where humanitarian projects were carried out. They raised awareness among the various stakeholders, well beyond their own organizations, and they contributed to pushing international organizations and researchers to further investigate the extent of the problem. Importantly, the need to overcome the ethically unacceptable divide between High-Income Countries and LMICs led pioneer “humanitarian pharmacists” to look for multidisciplinary collaborations, including with the academia. This was the case of Jacques Pinel, who actively disseminated the lessons from the field through different networks and tools, including peer-reviewed journals and academic textbooks [2, 9].

### Positive signals and persisting gaps

Some positive signals show that there is an increasing attention for the threats to individual and public health represented by poor-quality medicines. For instance, the World Health Organization (WHO) launched in 2001 the Prequalification (PQ) Programme, which had a major positive impact for assuring the quality of HIV/AIDS, malaria and tuberculosis medicines in LMICs [10]. The African Medicines Regulatory Harmonization Initiative was started in 2009 to increase access to safe and effective medicines of good quality [11], and the establishment of an African Medicines Agency is underway, “to ensure that all Africans have access to affordable medical products for priority diseases/conditions that meet internationally-recognized standards of quality, safety and efficacy” [12]. Other initiatives include QUAMED, a network of non-governmental organizations and sub-Saharan African procurement centers that pool resources for auditing distributors and manufacturers according to the WHO standards and share the gained knowledge [13]; the WWARN Medicine Quality Scientific Group, that shares expertise and collates information to increase understanding of the prevalence and distribution of poor-quality medicines [14]; and the joint project of the United States Pharmacopoeia and USAID to promote quality of medicines in developing countries, that strengthens quality assurance systems and quality control laboratories [15].

Importantly, the Sustainable Development Goal 3.8 aims at universal health coverage, including quality and affordable essential medicines and vaccines for all. To achieve this global aim, different interventions are needed at different levels, by broadening or replicating the positive initiatives listed above. Among others, the National Regulatory Authorities in LMICs should be strengthened, the information available on quality of medicines should be transparently shared, the WHO Prequalification Programme should be broadened to all the medicines included in the WHO Essential Medicines Model List, and the procurement policies of major donors and procurement agencies should be adapted to promote a uniform reference to WHO standards. But the process remains slow, and many communities are still exposed to avoidable risks, not only due to the illegal, falsified medicines, but especially due to legitimate sub-standards [7]. This reality is not limited to Sub-Saharan Africa. For instance, in Pakistan a case of cross-contamination during the manufacturing process was discovered after the contaminated medicine caused the death of 120 patients [16]. A recent cross-sectional study carried out in China to investigate the content in active ingredient of five antimicrobial drugs found that all tested samples were registered in the country, but 15 % failed to meet the Chinese Pharmacopeia standard [17].

### The role of advocacy and pedagogy

Despite the increasing evidence that weaknesses in pharmaceutical quality assurance have been causing a great deal of avoidable morbidity and mortality, awareness remains quite low among non-specialists, including academics and policy makers. For instance, substandard antibiotics are likely to be a powerful contributor to the emergence of resistances, since they may be under-dosed or poorly bioavailable, resulting in sub-therapeutic doses. However, their role is frequently downplayed in policy documents concerning the global fight against antimicrobial resistance, e.g. the list of interventions proposed in the recent report “Tackling drug-resistant infections globally: final recommendations” [18] does not include a specific action concerning the fight against poor-quality antibiotics. We formulate the hypothesis that this is at least partly due to the fact that pharmaceutical regulation is still insufficiently or inadequately addressed in the study curricula of future policy makers. Thus, also the deleterious consequences of poor pharmaceutical regulation on health systems and public health tend to be neglected.

In this scenario, more and broader advocacy is needed for universal access to quality-assured medicines, targeting all those who are differently “involved” with medicines: regulators, international organizations, journalists, purchasers, prescribers, program managers, policy makers, public health actors and, obviously, those who are directly exposed to the consequences of inadequate practices and policies: the patients.

Advocacy should be based on strong evidence from research and monitoring programs. A lot has been published, even recently [19–22], but the methodology is not always consistent across studies, and some geographic regions have been neglected by the researchers [5]. On a positive note, the WHO recently published new guidelines on the conduct of surveys of the quality of medicines [23], which provide National Regulatory Authorities, international organizations, procurement agents, nongovernmental organizations and academic or research groups with a robust methodology. Results of well-conducted surveys will help to better evaluate the prevalence of poor-quality medicines in specific countries/regions, to identify the specific weaknesses along the supply chain, and to design tailored corrective actions. In addition, by documenting the extent of the problem, these studies would provide an important evidence-based support for advocacy.

But academic articles and technical paper can be difficult to understand for non-specialists. Adequate communication tools should be developed, in lay language, to address those who do not have a technical background in quality assurance of medicines, but may play an important role in defining policies and/or in advocating for universal access to quality-assured medicines. Jacques Pinel, who passed away in 2015, was acquainted with the difficulties of dialoguing with a variety of counterparts that rarely share the same technical knowledge. An important lesson that we should retain from his work [24] is that continuing education, and dissemination of information on pharmaceutical regulation and standards in a language comprehensible to all, are essential for an effective advocacy in the fight against the epidemic of poor-quality medicines in LMICs. The importance of giving accurate information in lay language is demonstrated by the experience of the advocates of access to medicines. They managed to make complex intellectual property issues understandable by the scientific and international community, so creating a powerful advocacy movement which has been able to trigger important changes [25].

## Conclusion

The development and dissemination of communication tools for explaining in lay language the meaning of "medicines quality", the factors enhancing or hindering it, and the consequences of poor-quality medicines for individual and public health, is needed to foster advocacy and to create a favorable environment for policy changes. The fight to ensure universal access to quality medicines needs the participation of all, and can only be successful if grounded in common understanding.

## Abbreviations

LMICs: Low- and middle-income countries
PQ: Pre-qualification programme
SSFFC: Substandard, spurious, falsely labelled, falsified and counterfeit
WHO: World Health Organization.
